# Development of a personal competency evaluation indicator system for tuberculosis health educators: a scoping review and Delphi study

**DOI:** 10.3389/fpubh.2026.1861732

**Published:** 2026-07-08

**Authors:** Xu Song, Qingjun Jia, Qingchun Li, Yinyan Huang, Ruoqi Dai, Qinglin Cheng

**Affiliations:** Department of Tuberculosis Control and Prevention, Hangzhou Center for Disease Control and Prevention (Hangzhou Health Supervision Institution), Hangzhou, China

**Keywords:** analytic hierarchy process, competency evaluation, Delphi method, health education, scoping review, tuberculosis

## Abstract

**Objective:**

To systematically synthesize evidence on the personal competency of tuberculosis (TB) health educators and to develop a scientifically robust and comprehensive evaluation indicator system.

**Methods:**

A two-phase mixed-methods design was employed. Phase 1 involved a scoping review of seven databases (PubMed/MEDLINE, Web of Science, Scopus, Embase, CINAHL, ERIC, and the Cochrane Library) to identify competency-related literature on TB health educators, with no restrictions on publication date, language, or geographic setting. Extracted competency elements were synthesized into a preliminary framework. Phase 2 used a modified Delphi method with two rounds of consultation among 20 Chinese experts in TB control and health education. Indicator importance was rated on a 5-point Likert scale, and the Analytic Hierarchy Process (AHP) was applied to calculate indicator weights. Validity and reliability were assessed using content validity indices and intraclass correlation coefficient (ICC).

**Results:**

The scoping review included 36 studies, from which seven core dimensions, 24 categories, and 63 specific competency elements were extracted. Both Delphi rounds achieved a 100% response rate. The expert authority coefficient (Cr) was 0.84. Kendall’s W increased significantly from 0.295 (first round) to 0.328 (second round, both *p* < 0.001). After the second round, all indicators met the retention criteria: mean importance ≥3.5, coefficient of variation <0.25, and expert agreement rate ≥50.0%. AHP results showed good consistency (first-level indicator CR = 0.043; all second-level CRs < 0.10). The overall scale demonstrated good reliability (ICC = 0.86) and content validity (scale-level CVI = 0.92; item-level CVI range: 0.85–1.00). The highest-weighted dimension was Communication and Health Education Skills (0.235).

**Conclusion:**

The personal competency evaluation indicator system for TB health educators developed in this study is scientifically sound, reliable, and practical. It provides a validated tool for competency assessment, targeted training design, and workforce development in TB health education.

## 1 Introduction

Tuberculosis (TB) remains one of the world’s deadliest infectious diseases. The World Health Organization’s Global Tuberculosis Report 2025 indicates that in 2024, there were approximately 10.7 million new TB cases and 1.23 million TB-related deaths globally (1). In the context of the End TB Strategy, health education plays an irreplaceable role as a crucial intervention in TB prevention and control, improving patient treatment adherence, promoting early detection and diagnosis, and reducing social discrimination (2). Recent field-based evidence has further demonstrated that well-designed TB health education programs can significantly reduce patient dropout rates and improve treatment completion (3). However, studies also reveal persistent gaps in patient understanding of TB, widespread public misconceptions about its transmission, and the negative impact of TB-related stigma on health-seeking behavior (4, 5). These findings underscore the need for competent health educators who can address these barriers effectively.

Health educators are key human resources in the TB control system. They are responsible for patient education, community mobilization, health counseling, and follow-up management. Their personal competency directly affects the effectiveness of health education and, consequently, TB control outcomes (6–8). However, current research on the personal competency of TB health educators is fragmented in three main aspects: (a) inconsistent competency dimensions across studies, with some focusing only on knowledge or attitudes rather than a comprehensive set; (b) target-group specific frameworks (e.g., only for nurses or community health workers) that are not easily generalizable; and (c) lack of agreement on which specific skills are most critical for effective TB health education. This fragmentation leads to unfocused training and inconsistent evaluation standards in real practice—for instance, training programs may omit essential skills, and performance assessments vary widely across institutions, making it difficult to benchmark or improve educator quality. There is a lack of a unified and systematic competency evaluation framework. Existing studies mostly focus on single dimensions of knowledge, attitude, and practice (KAP) (4, 5, 9) or describe capacities for specific types of workers (e.g., community health workers, nurses) (10–13). A comprehensive, multi-level, and operational competency evaluation system has yet to be established.

Developing a scientific competency evaluation system is crucial for defining job competency requirements for TB health educators, conducting targeted training, evaluating work performance, and promoting workforce development (14, 15). This study aims to systematically synthesize research on the personal competency of TB health educators through a scoping review, construct a competency evaluation indicator system using the Delphi method, and determine indicator weights using AHP, thereby providing a scientific tool for competency assessment.

## 2 Materials and methods

### 2.1 Phase 1: scoping review

The first phase of this study followed the five-stage framework proposed by Arksey and O’Malley (16): (1) identifying the research question, (2) identifying relevant studies, (3) study selection, (4) charting the data and (5) collating, summarizing, and reporting the results. The scoping review was considered appropriate for mapping the content, scope, breadth, nature, and evolution of existing evidence on TB health educator competency. This study adhered to the Arksey and O’Malley framework (16), which does not mandate registration.

#### 2.1.1 Identifying the research question

This scoping review addressed the following question: What concepts, dimensions, or specific elements related to the personal competency of TB health educators are described in the existing literature? A preliminary search of the Cochrane Database was conducted to identify existing systematic reviews on this topic; none were found.

#### 2.1.2 Inclusion criteria

The inclusion criteria focused on the personal competency of TB health educators. To comprehensively present the content of competency assessment for TB health educators, no restrictions were placed on geographic location, publication date, or language. Following the JBI Participants, Concept, and Context (PCC) framework, the following inclusion criteria were established:

Participants: All TB health educators, including full-time and part-time workers, regardless of gender, ethnicity, or economic status. These include but are not limited to physicians, nurses, and community health workers.
Concept: Literature reporting content related to the personal competency assessment of TB health education, including influencing factors.
Context: No restrictions on study setting, including communities, hospitals, CDCs, health administrative departments, outpatient clinics, inpatient wards, or any country or region where health education activities occur.
Types of evidence sources: Any existing literature, such as original research articles, systematic reviews, meta-analyses, letters, and guidelines.

#### 2.1.3 Exclusion criteria

Studies where the full text could not be obtained and duplicate publications were excluded. In addition, non-empirical studies (e.g., conference abstracts, editorials, popular science articles) were excluded.

#### 2.1.4 Information sources

A preliminary search was initially conducted in MEDLINE (EBSCO) and CINAHL (EBSCO) to identify relevant articles and key terms. Key terms were matched with MeSH terms, and a search string combining MeSH terms and MEDLINE keywords was developed. The search strategy was adapted for each information source, including all identified keywords and index terms (Appendix I). The comprehensive databases searched were PubMed/MEDLINE, Web of Science Core Collection, Scopus, Embase, CINAHL, ERIC (EBSCOhost), and the Cochrane Library.

#### 2.1.5 Search

The preliminary search strategy was pilot-tested and used to guide the development of database-specific strategies. The search combined four main concepts – tuberculosis, health education, health worker, and competency – with their synonyms using Boolean operators “AND” and “OR.” To ensure precision, we used phrase searching (e.g., “health education” in double quotes), truncation (e.g., competen* to capture competency, competence), and field-specific limits (e.g., title/abstract) where applicable. The detailed search strings, including all synonyms and database-specific syntax, are provided in Appendix I. The search was conducted progressively across databases, with the final search completion date set as October 14, 2025.

#### 2.1.6 Selection of evidence sources

Initial searches indicated that the research strategy retrieved a large number of irrelevant records. However, to systematically map the scope, coverage, nature, and development of existing literature on TB health educator personal competency, the research team opted for a broad exploratory strategy rather than a focused one. A comprehensive search strategy was employed to minimize the risk of missing relevant literature. Retrieved records were first imported into EndNote 21.3 for duplicate checking and then transferred to Rayyan (17) for blinded screening. Two reviewers independently screened titles, abstracts, and full texts to determine eligibility. To assess inter-rater consistency, we calculated Cohen’s kappa coefficient for both the title/abstract screening and full-text screening stages. Disagreements were resolved by two independent research team members evaluating the full text.

#### 2.1.7 Data charting process

One research team member performed data charting, while two other members independently verified the charted data. A mixed-methods data charting form, including general and methodological information, was piloted on a small sample of included studies to ensure systematic capture of all key data.

#### 2.1.8 Critical appraisal of individual sources of evidence

As this scoping review followed the five-stage framework of Arksey and O’Malley (16), a critical appraisal of the methodological quality or potential bias of individual evidence sources was not performed.

#### 2.1.9 Synthesis of results

We used a thematic synthesis approach to aggregate the extracted competency elements into a structured framework. Two reviewers independently coded the extracted elements from the included studies using an open-coding method. Each code represented a distinct competency-related concept. The two reviewers then independently grouped the codes into preliminary categories based on conceptual similarity. After independent grouping, the reviewers met to compare their categorizations. Disagreements were resolved through discussion; if consensus could not be reached, a third reviewer was consulted. The resulting categories were then iteratively reviewed and clustered into broader themes. This process was repeated until a stable hierarchical structure emerged. The entire research team, which included experts from CDCs and hospitals, reviewed and discussed the framework to ensure its content validity and practical relevance. Any remaining disagreements were resolved by group discussion.

### 2.2 Phase 2: Delphi study

The Delphi method is a structured process for achieving consensus among a panel of experts on a specific topic through multiple iterative rounds (18, 19).

#### 2.2.1 Questionnaire composition and indicator scoring

The questionnaire comprised three parts: instructions, an indicator importance assessment form, and an expert demographic information form. 1. The instructions introduced the study purpose, completion guidelines, and contact information. 2. The indicator importance assessment form included dimension lists and importance rating scales. Importance was rated using a 5-point Likert scale (5 = extremely important, 4 = important, 3 = neutral, 2 = unimportant, 1 = extremely unimportant). The mean score for each indicator represents its importance. 3. The expert information form collected demographics, level of knowledge about the topic, and the basis for their judgments.

#### 2.2.2 Expert selection criteria

Experts were required to have long-term experience in TB prevention, treatment, or health education, a bachelor’s degree or higher, at least 10 years of work experience, and a high level of motivation to participate in the indicator development process.

#### 2.2.3 Expert consultation

Electronic questionnaires were used for two rounds of expert consultation. After the first round, expert opinions were analyzed and used to modify the questionnaire before the second round.

#### 2.2.4 Indicator selection criteria

Indicators were retained if they met the following criteria: (1) mean importance score ≥ 3.5, coefficient of variation (CV) ≤ 0.25, and expert agreement rate ≥ 50%; or (2) they were identified through group discussion based on expert comments.

#### 2.2.5 Quality control

During the design phase, Chinese and international literature was reviewed to understand the current state of research, and experts in the field were consulted to ensure the feasibility and validity of the research methods. During the implementation phase, experts were strictly selected according to the criteria, data from assessment forms were accurately recorded and subjected to reliability and validity analysis, and data collection quality was checked periodically. During the data entry phase, two individuals independently double-entered the consultation data.

#### 2.2.6 Data processing and statistical analysis

Data were entered using EpiData 3.1 and analyzed using SPSS 24.0. Statistical calculations included the expert positive coefficient, authority coefficient, degree of consensus, degree of coordination, and intraclass correlation coefficient (ICC). The expert positive coefficient was evaluated using the effective response rate. The expert authority coefficient (Cr) was determined by the familiarity coefficient (Cs) and judgment coefficient (Ca): Cr = (Ca + Cs)/2. Expert authority was measured using self-assessment with quantified values. The degree of consensus was evaluated using the mean importance score (M), CV, and agreement rate (N). The degree of expert coordination was expressed using Kendall’s W. YAAHP 12.11 software was used to calculate indicator weights and consistency ratios (CR) using AHP. The judgment matrices were constructed as follows: after the second Delphi round, the geometric mean of the 20 experts’ importance scores for each indicator was calculated. Pairwise comparisons between indicators at the same hierarchical level were then performed using the 1–9 Saaty scale, based on the ratio of their geometric means. The geometric mean matrix was input into YAAHP, which automatically computed the maximum eigenvalue (λmax), consistency index (CI), and consistency ratio (CR). The intraclass correlation coefficient (ICC) was used as a measure of inter-rater agreement. The significance level was set at *α* = 0.05.

## 3 Results

### 3.1 Phase 1: scoping review

#### 3.1.1 Study selection results

**Figure 1:**
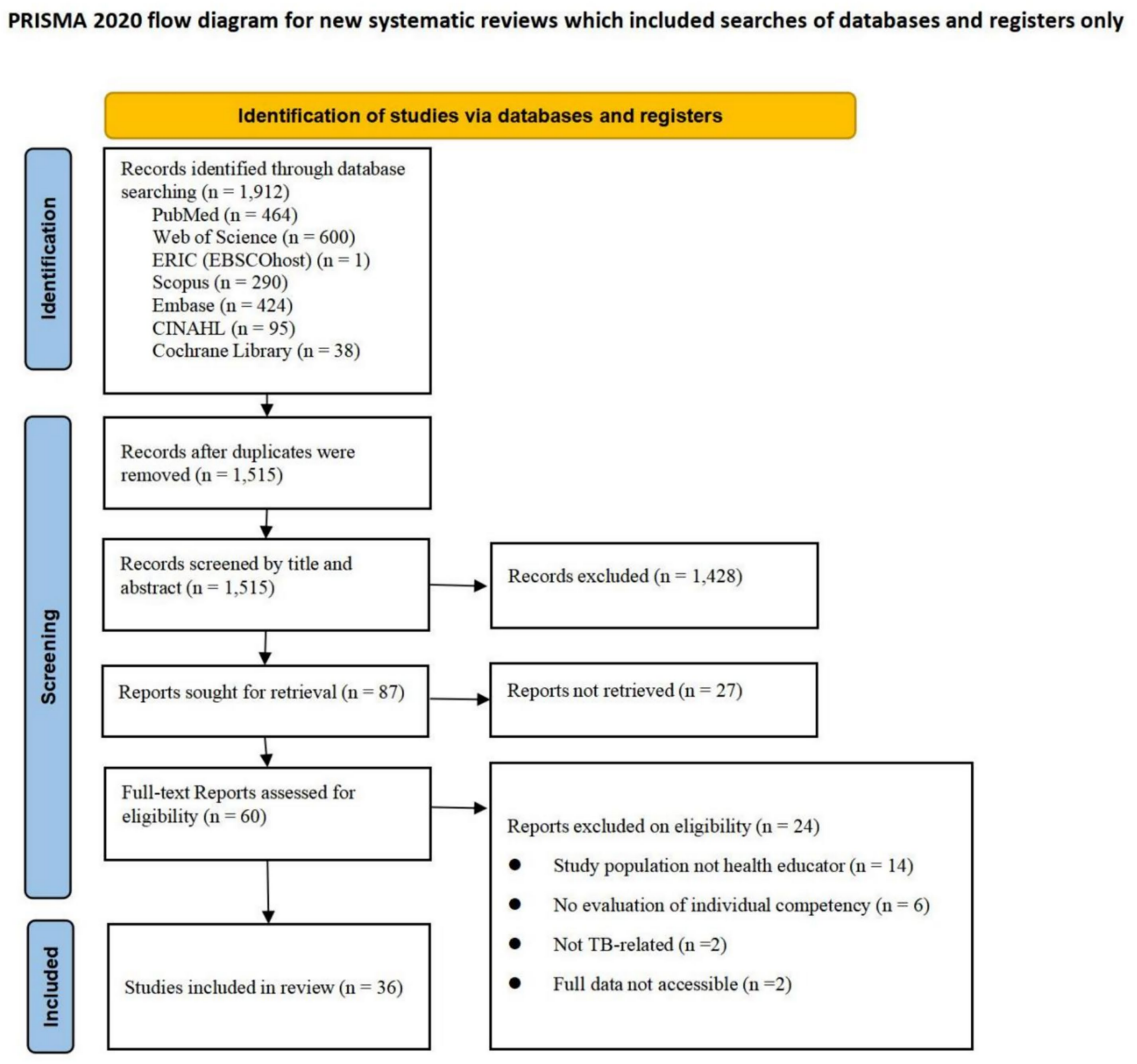
PRISMA flowchart.

The electronic search yielded 1,912 records. After deduplication, 1,515 records remained. After screening based on titles and abstracts, 1,428 records were excluded, and 87 full-text reports were assessed for eligibility. However, 27 of them could not be obtained. Ultimately, 60 full-text reports were assessed, of which 24 were excluded (Appendix III), and 36 studies (Appendix II) met the inclusion criteria. The study selection process is presented in the PRISMA flowchart (Figure 1). The inter-rater Cohen’s kappa coefficient was 0.84 (95% CI: 0.81–0.87) for title/abstract screening and 0.91 (95% CI: 0.88–0.94) for full-text screening.

#### 3.1.2 Characteristics of evidence sources

The publication years ranged from 1999 to 2025.

##### 3.1.2.1 Country distribution

**Figure 2:**
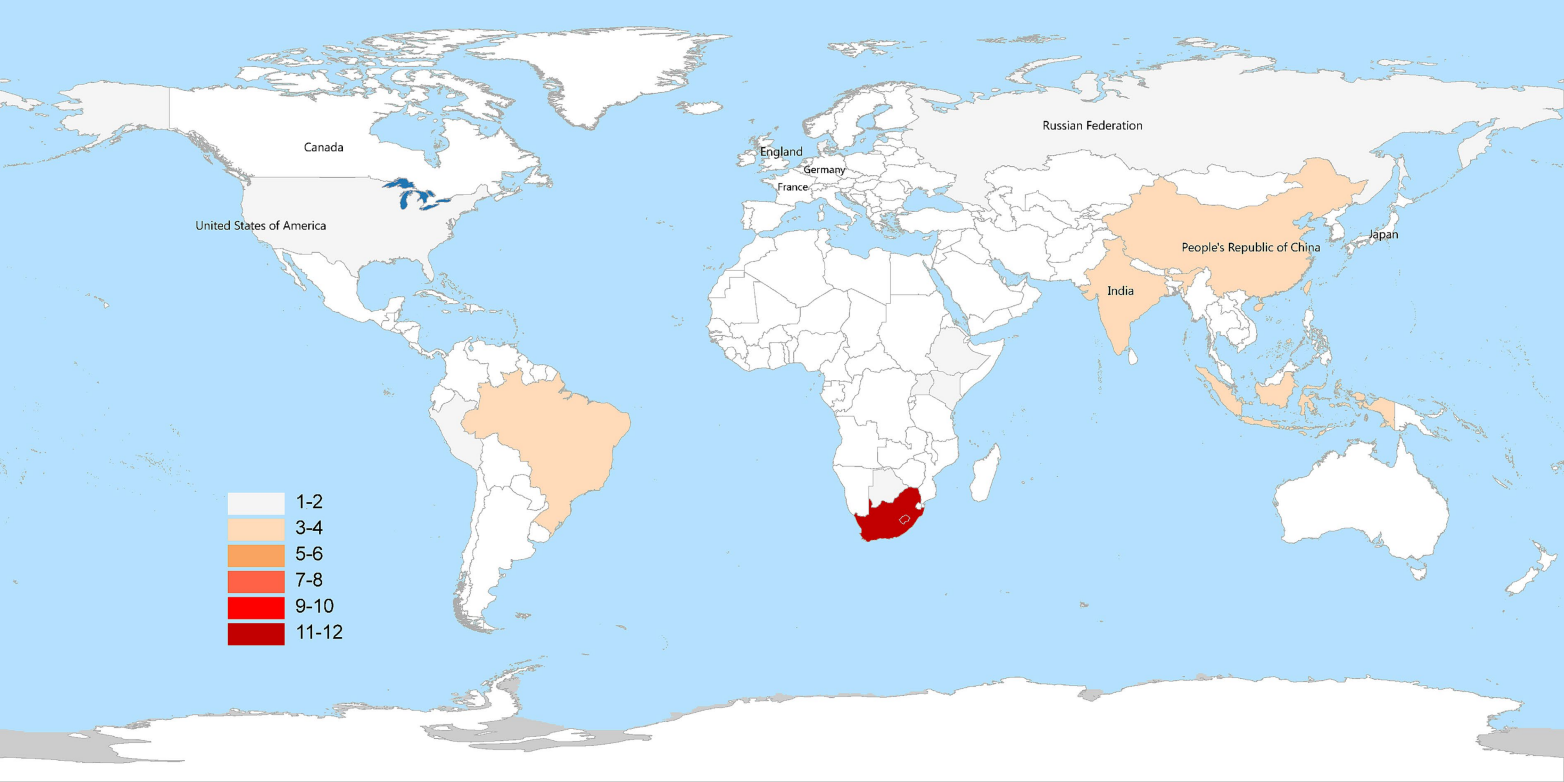
Geographical distribution of the included studies by country.

South Africa (*n* = 11), China (*n* = 4), India (*n* = 3), Brazil (*n* = 3), Indonesia (*n* = 3), Multinational/Global (*n* = 3), Denmark (*n* = 1), Botswana (*n* = 1), United States (*n* = 1), Haiti (*n* = 1), Kenya (*n* = 1), Uganda (*n* = 1), Russia (*n* = 1), Peru (*n* = 1), Ethiopia (*n* = 1) (Figure 2).

##### 3.1.2.2 Participant types

Included healthcare workers, community health workers, nurses, doctors, village doctors, pharmacy staff, medical students, residents, home care providers, community health volunteers, and academic-practitioners.

##### 3.1.2.3 Data collection methods

Included questionnaires, in-depth interviews, focus group discussions, semi-structured interviews, video recordings, role-playing, rating scales, medical record reviews, social network analysis, and literature searches.

#### 3.1.3 Synthesis of results

Following integration, the personal competency of TB health educators was organized into seven main dimensions (Appendix IV):

(1) Professional Knowledge Base; (2) Communication and Health Education Skills; (3) Clinical Practice and Problem-Solving Skills; (4) Professionalism and Personal Attributes; (5) Training, Learning, and Development competency; (6) Collaboration, Management, and Resource Integration competency; (7) Research and Knowledge Translation Competence.

The Professional Knowledge Base included core TB knowledge and related domain knowledge. Core TB knowledge comprised: basic TB knowledge (etiology, transmission routes, affected organs); mastery of TB diagnostic criteria and procedures; identification of TB risk factors; knowledge of TB drugs (types, mechanisms, side effects); and TB treatment regimens (course, duration). Related domain knowledge included: ability to integrate cross-disciplinary knowledge; knowledge of disease associations (comorbidities, complications); and understanding of program operations and health policies.

Communication and Health Education Skills included core communication skills, health education implementation skills, special situation communication skills, and educational methods/tools application. Core communication skills comprised: general communication techniques, active listening, rapport building, non-judgmental communication, empathy and emotional support, psychological support, and patient validation. Health education implementation skills included: patient education ability, ability to develop health education plans, ability to identify health education needs, information presentation and delivery, and cognitive verification skills. Special situation communication skills included: ability to overcome barriers in discussing sensitive topics, motivational interviewing skills, and stigma response and anti-discrimination communication skills. Educational methods/tools application included: ability to use multimedia/digital tools, ability to organize meetings and activities, and ability to apply tools and adapt to context.

Clinical Practice and Problem-Solving Skills included clinical practice skills, problem-solving skills, and follow-up/case management skills. Clinical practice skills comprised: clinical decision-making, general practical operation skills, symptom recognition, and infection control implementation. Problem-solving skills included: general problem-solving, practical problem analysis, flexibility and adaptability, and reflection and improvement skills. Follow-up/case management skills included: follow-up and tracking skills, confidentiality management, and ability to respond to patient needs.

Professionalism and Personal Attributes included professional values and motivation, attitudes and affective traits, self-awareness and management, bias and stigma management, and key personality traits. Professional values and motivation included: sense of professional and ethical responsibility, sense of mission, and motivation for self-improvement. Attitudes and affective traits included: positive work attitude, empathy and altruism, sense of achievement and pride, and work initiative. Self-awareness and management included: self-confidence, clarity of role perception, self-emotion management, self-care, and emotional stability. Bias and stigma management included: stigma awareness and anti-stigma literacy, and ability to maintain objectivity. Key personality traits included: general personality traits (e.g., openness, conscientiousness).

Training, Learning, and Development competency included training experiences and formats, learning and development ability, and teaching and dissemination ability. Training experiences and formats included: formal training experience, continuous training participation, standardized/specialized training, distance education, and specific communication skills training. Learning and development ability included: active learning ability, continuing education participation, knowledge updating and continuous learning, and knowledge retention/updating. Teaching and dissemination ability included: teaching and training ability, knowledge transfer skills, and public education ability.

Collaboration, Management, and Resource Integration competency included teamwork ability, resource integration and referral, cross-sectoral coordination, community mobilization, and organizational and management ability. Teamwork ability comprised: communication and collaboration ability, professional consultation and collaboration, collaborative awareness, and networking ability. Resource integration and referral included: resource linkage and referral ability. Cross-sectoral coordination included: cross-service communication and coordination ability, and task sharing and task shifting ability. Community mobilization included: community mobilization ability and community trust-building ability. Organizational/management ability included: relationship management and communication environment creation, and communication and persuasion skills.

Research and knowledge translation competence included research ability, knowledge translation ability, and project innovation ability. Research ability comprised: general knowledge and research skills, operational research skills. Knowledge translation ability included: knowledge translation ability. Project innovation ability included: project innovation ability and targeted intervention design ability.

### 3.2 Phase 2: Delphi study

#### 3.2.1 Expert demographic information

Among the 20 selected experts, 17 (85.0%) worked in TB prevention and control at CDCs, and 3 (15.0%) worked in health education at CDCs. Six experts (30.0%) held senior professional titles, 12 (60.0%) held deputy senior titles, and 2 (10.0%) held intermediate titles. All had a bachelor’s degree or higher and at least 10 years of work experience.

#### 3.2.2 Expert positive coefficient

Two rounds of questionnaires were sent to the 20 experts. All 20 experts responded in both rounds, yielding a 100% effective response rate for each round.

#### 3.2.3 Expert authority coefficient

The results showed an authority coefficient (Cr) of 0.84.

#### 3.2.4 Degree of expert consensus

After the second round, the mean importance scores of all indicators were ≥3.5. The coefficients of variation ranged from 0.080 to 0.211, all <0.25. The expert agreement rate was ≥50.0%.

#### 3.2.5 Degree of expert coordination

After the first round, Kendall’s W was 0.295 (*p* < 0.001). After the second round, Kendall’s W increased to 0.328 (*p* < 0.001), indicating a statistically significant improvement in coordination. Although a Kendall’s W of 0.328 is generally considered moderate, it is acceptable given the large number of indicators (63 elements). The statistically significant increase from round 1 to round 2, together with the fact that all indicators met the retention criteria, confirms that acceptable consensus was achieved.

#### 3.2.6 Changes in evaluation indicators

##### 3.2.6.1 First round

The competency framework was optimized from 7 dimensions, 25 categories, and 80 competency elements to 7 dimensions, 24 categories, and 63 competency elements, laying the foundation for the second round of the Delphi study. The main revisions included:

(1) Addressing conceptual overlap: “empathy/emotional support” and “empathy/altruism” were split into “empathy expression ability” and “altruistic spirit”; broad items such as “general communication techniques” were removed, and “clear expression ability” was added.
(2) Adjusting subsumption relationships: TB-related knowledge was consolidated under the category “TB medical knowledge”; “understanding of program operations and health policies” was split into two categories: policy and health system knowledge.
(3) Eliminating tautology: Redundant items such as “patient education ability” and “general problem-solving ability” were removed.
(4) Integrating highly overlapping items: “Bias and stigma management” and related content in communication skills were merged into “stigma response communication ability.”
(5) Clarifying vague wording: “Drug knowledge” was broken down into “knowledge of side effects” and “knowledge of treatment regimens”; “patient needs response ability” was expanded to “individualized intervention design ability.”
(6) Removing less practical items: For example, “distance education,” “personality traits,” and “teaching and dissemination ability” were deleted.
(7) Adding practice-oriented items: Such as “ability to develop health education materials,” “stress coping ability,” and “experience summarization ability.” Results after the first round are presented in Appendix V.

##### 3.2.6.2 Second round

The competency framework remained unchanged in terms of 7 dimensions, 24 categories, and 63 specific competency elements, but the wording of some indicators was refined: “knowledge application ability” was expanded to “knowledge application and translation ability”; “self-care ability” was refined to “self-care and burnout prevention ability”; “literature review ability” was revised to “literature review and comprehension ability”; and “community trust-building ability” was expanded to “community relationship building and maintenance ability.” Final results after the second round are presented in Table 1.

**Table 1:** Competency evaluation index system and weights for tuberculosis health educators.

Dimension	Dimension weight	Category	Category weight	Specific competency element	Indicator weight
1 Professional knowledge base	0.186	1.1 Medical knowledge of tuberculosis	0.102	1.1.1 Tuberculosis etiology and transmission mechanisms	0.023
				1.1.2 Tuberculosis clinical manifestations and classification	0.022
				1.1.3 Tuberculosis diagnostic criteria and procedures	0.024
				1.1.4 Knowledge of tuberculosis treatment regimens	0.019
				1.1.5 Knowledge of anti-tuberculosis drug side effects	0.014
		1.2 Epidemiological knowledge	0.046	1.2.1 Pulmonary tuberculosis risk factor identification	0.024
				1.2.2 Awareness of tuberculosis epidemiological characteristics	0.022
		1.3 Policy and system knowledge	0.037	1.3.1 National tuberculosis prevention and control policies	0.020
				1.3.2 Awareness of the health service system	0.017
2 Communication and health education skills	0.235	2.1 Foundational communication skills	0.083	2.1.1 Active listening ability	0.025
				2.1.2 Non-judgmental communication ability	0.022
				2.1.3 Empathy expression ability	0.020
				2.1.4 Clear expression ability	0.016
		2.2 Health education implementation skills	0.071	2.2.1 Health education needs assessment ability	0.020
				2.2.2 Health education planning ability	0.018
				2.2.3 Health education material development and selection ability	0.017
				2.2.4 Educational effectiveness evaluation ability	0.016
		2.3 Special situation communication skills	0.051	2.3.1 Sensitive topic communication ability	0.014
				2.3.2 Motivational interviewing skills	0.013
				2.3.3 Psychological support and counseling ability	0.012
				2.3.4 Stigma response communication ability	0.011
		2.4 Educational forms and methods	0.031	2.4.1 Multimedia tool application ability	0.018
				2.4.2 Group activity organization and facilitation ability	0.013
3 Clinical practice and problem-solving skills	0.152	3.1 Clinical practice skills	0.065	3.1.1 Symptom recognition and preliminary judgment ability	0.023
				3.1.2 Infection control implementation ability	0.022
				3.1.3 Follow-up management ability	0.020
		3.2 Problem-solving skills	0.055	3.2.1 Practical problem analysis ability	0.020
				3.2.2 Emergency response ability	0.018
				3.2.3 Self-reflection and continuous improvement ability	0.017
		3.3 Case management ability	0.033	3.3.1 Confidentiality management ability	0.017
				3.3.2 Individualized intervention adjustment ability	0.016
4 Professionalism and self-management	0.123	4.1 Professional values	0.051	4.1.1 Sense of professional responsibility	0.018
				4.1.2 Sense of mission	0.017
				4.1.3 Altruistic spirit	0.016
		4.2 Work attitude	0.040	4.2.1 Proactivity	0.021
				4.2.2 Work enthusiasm and dedication	0.019
		4.3 Self-awareness	0.019	4.3.1 Clarity of role perception	0.010
				4.3.2 Professional Self-confidence	0.009
		4.4 Self-management ability	0.014	4.4.1 Emotional management ability	0.005
				4.4.2 Stress coping ability	0.005
				4.4.3 Self-care and burnout prevention ability	0.004
5 Learning and development competency	0.109	5.1 Learning behavior	0.045	5.1.1 Active learning behavior	0.024
				5.1.2 Continuous learning habit	0.021
		5.2 Training participation	0.035	5.2.1 Formal training experience	0.013
				5.2.2 Continuing education engagement	0.012
				5.2.3 Specialized skills training experience	0.011
		5.3 Knowledge management ability	0.029	5.3.1 Knowledge acquisition ability	0.010
				5.3.2 Knowledge update ability	0.010
				5.3.3 Knowledge application and translation ability	0.009
6 Collaboration and resource integration ability	0.102	6.1 Team collaboration ability	0.039	6.1.1 Teamwork ability	0.021
				6.1.2 Interprofessional collaboration ability	0.019
		6.2 Resource integration ability	0.028	6.2.1 Referral ability	0.014
				6.2.2 Social RESOURCE LINKING ABILITY	0.013
		6.3 Cross-sector coordination ability	0.020	6.3.1 Cross-institutional communication and coordination ability	0.011
				6.3.2 Task handover and coordination ability	0.010
		6.4 Community engagement ability	0.015	6.4.1 Community mobilization ability	0.008
				6.4.2 Community relationship building and maintenance ability	0.007
7 Research and innovation competence	0.093	7.1 Fundamental research ability	0.038	7.1.1 Literature review and comprehension ability	0.020
				7.1.2 Work data collection ability	0.018
		7.2 Knowledge translation ability	0.033	7.2.1 Evidence-based practice ability	0.017
				7.2.2 Experience summarization and refinement ability	0.016
		7.3 Innovation and improvement ability	0.022	7.3.1 Work problem identification and improvement ability	0.011
				7.3.2 Health education intervention design ability	0.010

#### 3.2.7 Weight calculation and consistency testing

The results of the weight calculation are shown in Table 1. Consistency testing results showed a CR of 0.043 for the first-level indicators and CRs ranging from 0.001 to 0.074 for the second-level indicators, all below 0.10. Among the seven dimensions, “Communication and Health Education Skills” had the highest global weight (0.235), followed by “Professional Knowledge Base” (0.186) and “Clinical Practice and Problem-Solving Skills” (0.152).

#### 3.2.8 Reliability and validity analysis

In the second round, 20 experts rated the importance of the final 63 indicators. Based on these ratings, the intraclass correlation coefficient (ICC) was 0.86 (95% CI: 0.79–0.91), indicating good inter-rater agreement.

Content validity analysis was used to examine whether the indicator items effectively reflected the measured variables. The proposed indicators were developed based on the synthesis of opinions from multiple experts in TB and health education, revised iteratively, thus demonstrating high content validity. The S-CVI was 0.92, and the I-CVI ranged from 0.85 to 1.00.

## 4 Discussion

### 4.1 Structural characteristics and theoretical connotation of the competency indicator system for TB health educators

**Figure 3:**
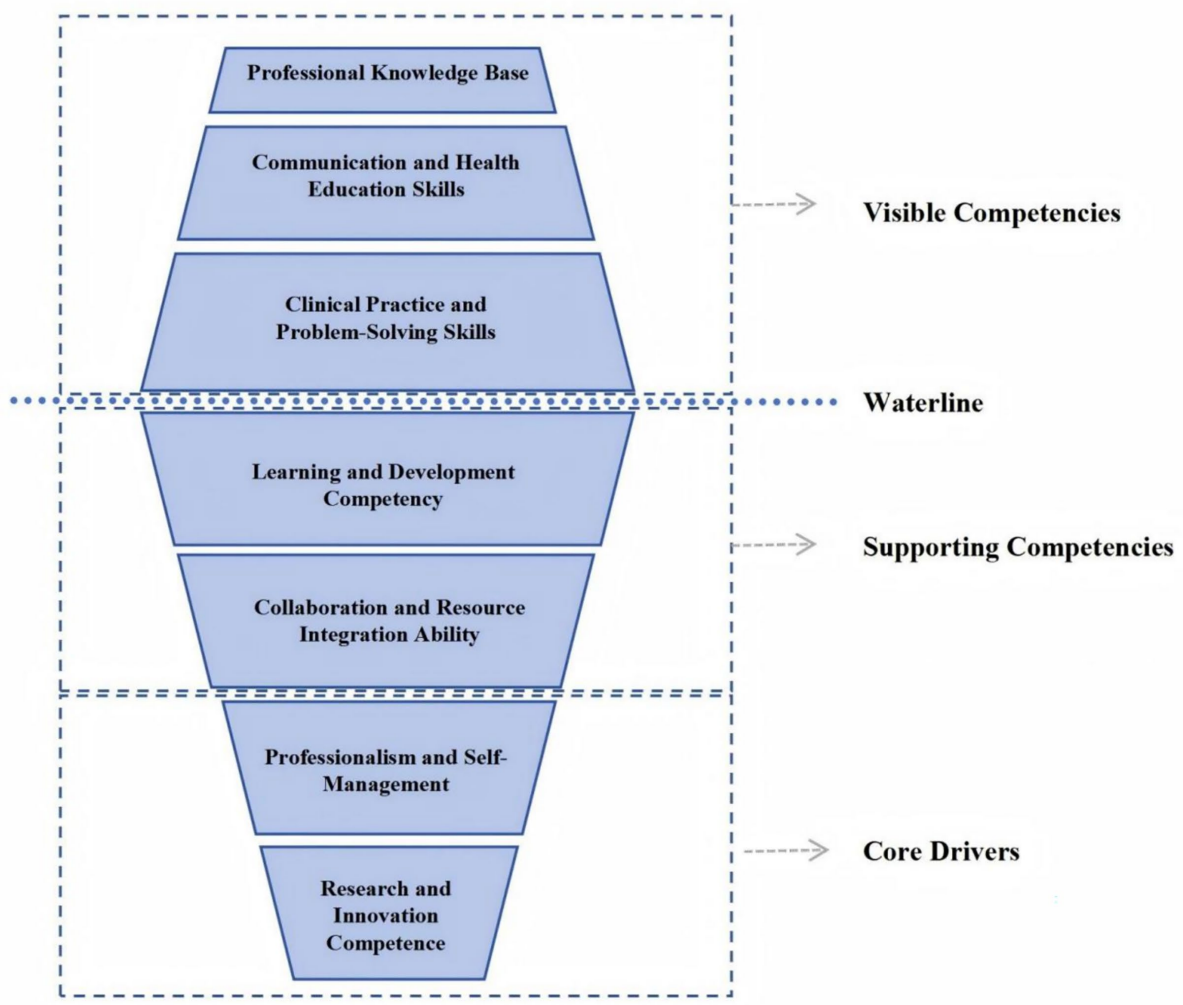
Iceberg competency model diagram.

The competency indicator system for TB health educators developed in this study comprises 7 dimensions, 24 categories, and 63 specific competency elements. According to the Iceberg Competency Model, this system can be organized into three levels (Figure 3).

The upper level of the iceberg comprises visible competencies, including Professional Knowledge Base, Communication and Health Education Skills, and Clinical Practice and Problem-Solving Skills. These competencies are directly observable and measurable, forming the foundational tools for health education. Notably, Communication and Health Education Skills carried the highest weight, aligning closely with the core responsibilities of health educators.

The middle level of the iceberg comprises supporting competencies, including Learning and Development competency, and Collaboration and Resource Integration Ability. These serve as a bridge between visible competencies and deeper drivers, playing a crucial role in the continuous development and practical application of competency.

The lower, deeper level of the iceberg comprises core drivers, including Professionalism and Self-Management, and Research and Innovation Competence. Located at the deepest level, these are the most difficult to cultivate but the most enduring, determining the long-term development potential and work quality of health educators.

The dimensions exhibit progressive and mutually reinforcing relationships: professional knowledge provides content for communication education, communication skills deliver that knowledge, practical skills ground the competencies, professionalism provides sustained motivation, learning and development ensure competency updating, collaboration and integration expand application scenarios, and research and innovation drive system optimization. This multi-dimensional, three-level competency structure provides a systematic theoretical framework for competency evaluation, training design, and workforce development.

### 4.2 Scientific rigor and reliability of the competency indicator system for TB health educators

This study initially constructed a competency framework comprising 7 dimensions, 25 categories, and 80 elements based on a scoping review. After two rounds of Delphi expert consultation, a final indicator system consisting of 7 dimensions, 24 categories, and 63 elements was established. In the first round, experts provided open-ended comments that led to systematic revisions of the initial indicators, addressing issues such as conceptual overlap, unclear subsumption relationships, tautology, and vague wording. Less practical items were removed, and practice-oriented items were added. In the second round, experts further refined the wording of some indicators (e.g., “knowledge application ability” was expanded to “knowledge application and translation ability”), making the indicator system clearer and more operational.

The 20 experts participating in both rounds were all from CDC institutions, of whom 85.0% worked in TB prevention and control and 15.0% in health education; 90.0% held senior professional titles, and all experts had a bachelor’s degree or higher and at least 10 years of work experience, ensuring the authority of the consultation. The expert authority coefficient (Cr) was 0.84, indicating a high level of expert authority (20, 21). The effective response rate for both questionnaire rounds was 100%, demonstrating a high degree of expert positivity. After the second round, the mean importance score of all indicators was ≥3.5, the coefficients of variation were all <0.25, and the expert agreement rate was ≥50.0%, meeting the prespecified selection criteria. Kendall’s W increased from 0.295 (*p* < 0.001) in the first round to 0.328 (*p* < 0.001) in the second round, and the improvement in coordination was statistically significant, indicating that expert opinions converged (20, 22).

The analytic hierarchy process consistency test showed a CR of 0.043 for the first-level indicators and CRs ranging from 0.001 to 0.074 for the second-level indicators, all below 0.10, indicating good consistency in expert judgments of indicator importance and reliable weight allocation (23, 24). Based on the 20 importance ratings from the second round, the intraclass correlation coefficient (ICC) was calculated as 0.86 (95% CI: 0.79–0.91), indicating good inter-rater agreement for the total scale. Content validity analysis showed an S-CVI of 0.92 and an I-CVI ranging from 0.85 to 1.00, both exceeding recommended standards, suggesting that the indicators effectively reflect the measured constructs (19, 24).

### 4.3 Composition analysis of the competency indicator system for TB health educators

The weights of the seven core dimensions are: Communication and Health Education Skills (0.235), Professional Knowledge Base (0.186), Clinical Practice and Problem-Solving Skills (0.152), Professionalism and Self-Management (0.123), Learning and Development competency (0.109), Collaboration and Resource Integration Ability (0.102), and Research and Innovation Competence (0.093).

Communication and Health Education Skills ranks highest, consistent with its core role. Within this dimension, Foundational Communication Skills (combined weight 0.083) and Health Education Implementation Skills (0.071) contribute the most, with “Active Listening Ability” (0.025) being the single highest indicator. In Professional Knowledge Base, Medical Knowledge of Tuberculosis (0.102) dominates, followed by Epidemiological Knowledge (0.046) and Policy and System Knowledge (0.037), reflecting the need for a comprehensive knowledge base. For Clinical Practice and Problem-Solving Skills, Clinical Practice Skills (0.065) and Problem-Solving Skills (0.055) are equally important. In Professionalism and Self-Management, Professional Values (0.051) far outweigh Self-Management Ability (0.014), indicating that experts value intrinsic drivers more. For Learning and Development competency, “Active Learning Behavior” (0.024) and “Continuous Learning Habit” (0.021) have higher weights than training participation indicators, suggesting that self-directed learning is prioritized. In Collaboration and Resource Integration Ability, Team Collaboration Ability (0.039) is higher than Community Engagement Ability (0.015), reflecting a preference for internal collaboration. Research and Innovation Competence has the lowest weight, with “Literature Review and Comprehension Ability” (0.020) being the largest contributor.

In summary, the indicator system is characterized by communication skills as the core, professional knowledge as the foundation, practical skills as the support, and intrinsic attributes as the driver. The weight distribution is reasonable and provides a quantitative basis for competency evaluation and training.

### 4.4 Application value and significance of the indicator system

The developed indicator system has several application values:

First, it provides a scientific tool for competency assessment, covering core competencies with expert-validated weights, enabling quantitative assessment for personnel selection, job matching, and performance evaluation.

Second, it offers a reference for diagnosing training needs. Analyzing scores across dimensions can identify individual or group competency gaps, informing targeted training plans and improving resource utilization.

Third, it serves as a benchmark for evaluating competency-building effectiveness, allowing pre-post measurement of competency changes to objectively assess training program impacts.

Fourth, it supports talent development pathways. Based on competency level classification (e.g., excellent, good, average, competent, needs improvement), different development paths can be planned, promoting structured workforce development.

Despite these potential applications, practical feasibility remains to be addressed. We acknowledge that the current 63-indicator system has not yet been piloted. A reduced subset of high-weight indicators (e.g., the top 20 indicators with the highest global weights) could be tested as a rapid screening tool. Future validation should include piloting both the full version and the shortened subset with 30–50 TB health educators in real-world settings to assess completion time, user acceptability, and inter-rater reliability. Field validation is therefore a necessary next step, and we plan to conduct such feasibility studies in subsequent research.

### 4.5 Limitations and future directions

This study has several limitations. (1) Institutional bias. All Delphi experts were recruited from Chinese CDCs. While they are highly experienced, a single-institution panel may overemphasize managerial and public health perspectives while underrepresenting clinical, community-based, or patient-centered views. Therefore, the indicator system’s validity in hospitals, primary care facilities, and cross-cultural contexts requires further testing. (2) Scoping review limitations. Following the Arksey & O’Malley framework, we did not perform quality appraisal of included studies – a common feature of scoping reviews. Consequently, some original evidence may have methodological weaknesses (e.g., selection bias, inadequate confounding control). In addition, 27 out of 87 full-text reports (31.0%) could not be retrieved despite attempts through interlibrary loan and contacting corresponding authors. This may introduce availability bias (e.g., non-English studies or negative findings might have been missed), and could have skewed the competency domains – for instance, missing perspectives from resource-limited settings or grassroots community health workers, which could have particularly affected competencies such as resource integration, community mobilization, and adaptation to low-resource contexts. (3) Practicality vs. indicator burden. The system comprises 63 specific indicators. The “practicality” claimed refers to its theoretical value for training needs assessment and job evaluation (theoretical practicality). However, 63 indicators may be cumbersome for routine use (operational practicality). These two aspects are not contradictory but reflect the transitional nature from a research tool toward a practice tool. A reduced subset of high-weight indicators could serve as a rapid screening tool, but this requires separate validation and pilot testing. (4) Cross-cultural applicability. The Delphi experts were all from Chinese CDCs, yet the scoping review included studies from South Africa, India, Brazil, Indonesia, and other countries – settings with markedly different TB burdens, health system structures, and cultural contexts. Chinese CDC experts may naturally prioritize competencies related to programmatic management, surveillance, and policy implementation, whereas settings with weaker health systems might emphasize community mobilization, patient adherence support, or task-shifting competencies. Therefore, while our indicator system is empirically grounded in a broad international literature, its weighting and specific wording reflect the Chinese expert perspective. Direct application of the system (especially the weight distribution) to other countries or health system contexts should be done with caution, and cultural/contextual adaptation is strongly recommended before use. Future multinational Delphi studies are needed to develop a globally adaptable core set of competencies. (5) AHP matrix construction. We constructed pairwise comparison matrices using the ratios of geometric means of Delphi importance scores rather than collecting separate pairwise judgments from each expert – the conventional AHP procedure. This deviation was adopted for feasibility: having 20 experts complete pairwise comparisons for 63 indicators would have required 1,953 judgments per expert (63 × 62/2), which is practically infeasible and would likely cause response fatigue. Our method leverages the Delphi consensus already achieved. However, it may not fully capture individual expert trade-offs between indicators. Future studies should cross-validate the weights using standard AHP on a reduced set of core indicators.

Future research should focus on: (1) conducting large-scale empirical studies to validate the reliability, validity, and operability of the system; (2) performing cross-cultural comparative studies to test applicability in different contexts; (3) exploring the association between competency scores and health education outcomes to validate effectiveness; and (4) developing supporting assessment tools and training resources to facilitate practical implementation.

## 5 Conclusion

This study developed a personal competency evaluation indicator system for TB health educators, comprising 7 dimensions, 24 categories, and 63 specific competency elements, using a scoping review and Delphi method. The system demonstrates good scientific rigor, reliability, and practicality. It can serve as a reference for the competency assessment, training, and workforce development of TB health educators.

## Data Availability

The original contributions presented in the study are included in the article/Supplementary material, further inquiries can be directed to the corresponding author.
