# Supplementary material for: Development of a personal competency evaluation indicator system for tuberculosis health educators: a scoping review and Delphi study
Source: Front Public Health. 2026 Jul 8;14:1861732. doi: 10.3389/fpubh.2026.1861732 (PMC13388549; doi:10.3389/fpubh.2026.1861732)
Supplement: Supplementary file 1 [file Data_Sheet_1.pdf]

Appendix I: Search strategy

PubMed

Search conducted: October 9, 2025

	Search	Results retrieved
1	("Tuberculosis"[Mesh] OR "Tuberculosis, Pulmonary"[Mesh] OR "Latent Tuberculosis"[Mesh] OR "Multidrug-Resistant Tuberculosis"[Mesh] OR TB[tiab] OR "Mycobacterium tuberculosis"[tiab] OR consumption[tiab] OR PTB[tiab])	716,880
2	("Health Education"[Mesh] OR "Health Promotion"[Mesh] OR "Patient Education as Topic"[Mesh] OR "Community Health Services"[Mesh] OR "health education"[tiab] OR "health promotion"[tiab] OR "patient education"[tiab] OR "community health"[tiab] OR "health communication"[tiab] OR "health literacy"[tiab] OR "behavior change communication"[tiab] OR BCC[tiab] OR "Information Education Communication"[tiab] OR IEC[tiab] OR "public health education"[tiab] OR "awareness campaign"[tiab] OR "health advoca*"[tiab] OR "health messag*"[tiab])	715,365
3	("Health Personnel"[Mesh] OR "Community Health Workers"[Mesh] OR "Allied Health Personnel"[Mesh] OR "Nurses"[Mesh] OR "Public Health Nursing"[Mesh] OR "health worker*"[tiab] OR "frontline worker*"[tiab] OR "lay health worker*"[tiab] OR "community health worker*"[tiab] OR "TB educator*"[tiab] OR "TB coordinator*"[tiab] OR "outreach worker*"[tiab] OR "field worker*"[tiab] OR counselor*[tiab] OR "human resources for health"[tiab] OR "health workforce"[tiab] OR "public health staff"[tiab] OR "healthcare provider*"[tiab] OR "health care provider*"[tiab])	788,427
4	("Professional Competence"[Mesh] OR "Clinical Competence"[Mesh] OR "Competency-Based Education"[Mesh] OR "Capacity Building"[Mesh] OR competenc*[tiab] OR capabilit*[tiab] OR skill*[tiab] OR "capacity building"[tiab] OR "knowledge attitude practice"[tiab] OR KAP[tiab] OR training[tiab] OR "skill development"[tiab] OR "performance improvement"[tiab] OR proficien*[tiab] OR qualification*[tiab] OR "core competenc*"[tiab] OR empower*[tiab] OR "ability"[tiab] OR "competency framework"[tiab] OR "skill set"[tiab])	2,517,821
5	1 and 2 and 3 and 4	464

Web of Science (Core Collection)

Search conducted: September 17, 2025

	Search	Results retrieved
1	(TS=("Tuberculosis" OR "TB" OR "Mycobacterium tuberculosis" OR "pulmonary TB" OR "PTB" OR "Latent TB" OR "MDR-TB" OR "XDR-TB" OR "consumption"))	1,243,128
2	(TS=("Health Education" OR "Health Promotion" OR "Patient Education" OR "health literacy" OR "Health Communication" OR "behavior change communication" OR "BCC" OR "Information Education Communication" OR "IEC" OR "public health education" OR "awareness campaign" OR "health advocac*" OR "health messag*" OR "community health education" OR "health coach*"))	267,512
3	(TS=("health personnel" OR "health worker*" OR "community health worker*" OR "frontline worker*" OR "lay health worker*" OR "nurse*" OR "public health nurse" OR "TB educator*" OR "TB coordinator*" OR "outreach worker*" OR "counselor*" OR "human resources for health" OR "health workforce" OR "healthcare provider*" OR "health care provider*" OR "allied health personnel" OR "staff" OR "personnel"))	716,577
4	(TS=("professional competence" OR "clinical competence" OR "competenc*" OR "capabilit*" OR "skill*" OR "capacity building" OR "knowledge attitude practice" OR "KAP" OR "training" OR "skill development" OR "performance improvement" OR "proficien*" OR "qualification*" OR "core competenc*" OR "empower*" OR "ability" OR "competency framework" OR "skill set" OR "capacity strengthening"))	3,934,040
5	1 and 2 and 3 and 4	600

Cochrane Library

Search conducted: October 9, 2025

	Search	Results retrieved
1	MeSH descriptor: [Tuberculosis] explode all trees	3,559
2	(Tuberculosis OR TB OR "Mycobacterium tuberculosis" OR "pulmonary TB" OR PTB OR "Latent TB" OR "MDR-TB" OR "XDR-TB"):ti,ab,kw	10,767
3	1 or 2	10,776
4	MeSH descriptor: [Health Education] explode all trees	26,768
5	("Health Education" OR "Health Promotion" OR "Patient Education" OR "health literacy" OR "Health Communication" OR "behavior change communication" OR BCC OR "Information Education Communication" OR IEC OR "public health education" OR "awareness campaign" OR (health NEXT advocac*) OR (health NEXT messag*) OR "community health education" OR (health NEXT coach*)):ti,ab,kw	50,131
6	4 or 5	52,814
7	MeSH descriptor: [Health Personnel] explode all trees	16,350
8	(("health personnel" OR (health NEXT worker*) OR (community NEXT health NEXT worker*) OR (frontline NEXT worker*) OR (lay NEXT health NEXT worker*) OR nurse* OR "public health nurse" OR (TB NEXT educator*) OR (TB NEXT coordinator*) OR (outreach NEXT worker*) OR (field NEXT worker*) OR counselor* OR "human resources for health" OR "health workforce" OR "public health staff" OR (healthcare NEXT provider*) OR (health NEXT care NEXT provider*) OR "allied health personnel" OR staff OR personnel)):ti,ab,kw	88,148

9	7 or 8	95,914
10	MeSH descriptor: [Professional Competence] explode all trees	5,798
11	(("professional competence" OR "clinical competence" OR competenc* OR capabilit* OR skill* OR "capacity building" OR "knowledge attitude practice" OR KAP OR training OR "skill development" OR "performance improvement" OR proficien* OR qualification* OR (core NEXT competenc*) OR empower* OR ability OR "competency framework" OR "skill set" OR "capacity strengthening")):ti,ab,kw	246,497
12	10 or 11	246,497
13	3 and 6 and 9 and 12	38

ERIC (EBSCOhost)

Search conducted: September 22, 2025

	**Search**	**Results retrieved**
1	(DE "Tuberculosis") OR (TI (Tuberculosis OR TB OR "Mycobacterium tuberculosis" OR "pulmonary TB" OR PTB OR "Latent TB" OR "MDR-TB" OR "XDR-TB") OR AB (Tuberculosis OR TB OR "Mycobacterium tuberculosis" OR "pulmonary TB" OR PTB OR "Latent TB" OR "MDR-TB" OR "XDR-TB"))	2,004
2	(DE "Health Education" OR DE "Health Promotion") OR (TI ("Health Education" OR "Health Promotion" OR "Patient Education" OR "health literacy" OR "Health Communication" OR "behavior change communication" OR BCC OR "Information Education Communication" OR IEC OR "awareness campaign" OR health NEXT advocac* OR health NEXT messag* OR "community health education") OR AB ("Health Education" OR "Health Promotion" OR "Patient Education" OR "health literacy" OR "Health Communication" OR "behavior change communication" OR BCC OR "Information Education Communication" OR IEC OR "awareness campaign" OR health NEXT advocac* OR health NEXT messag* OR "community health education"))	25,846
3	(DE "Health Personnel" OR DE "Community Health Services" OR DE "Nurses" OR DE "Teachers") OR (TI ("health personnel" OR health NEXT worker* OR community NEXT health NEXT worker* OR frontline NEXT worker* OR lay NEXT health NEXT worker* OR nurse* OR "public health nurse" OR TB NEXT educator* OR TB NEXT coordinator* OR outreach NEXT worker* OR counselor* OR "health workforce" OR trainer* OR facilitator*) OR AB ("health personnel" OR health NEXT worker* OR community NEXT health NEXT worker* OR frontline NEXT worker* OR lay NEXT health NEXT worker* OR nurse* OR "public health nurse" OR TB NEXT educator* OR TB NEXT coordinator* OR outreach NEXT worker* OR counselor* OR "health workforce" OR trainer* OR facilitator*))	74,384
4	(DE "Professional Competence" OR DE "Capacity Building" OR DE "Teacher Competencies" OR DE "Job Skills") OR (TI ("professional competence" OR competenc* OR skill* OR "capacity building" OR "knowledge attitude practice" OR KAP OR training OR "skill development" OR "performance improvement" OR proficien* OR qualification* OR core NEXT competenc* OR empower* OR "ability" OR "curriculum development" OR "training program*") OR AB ("professional competence" OR competenc* OR skill* OR "capacity building" OR "knowledge attitude practice" OR KAP OR training OR "skill development" OR "performance improvement" OR proficien* OR qualification* OR core NEXT competenc* OR empower* OR "ability" OR "curriculum development" OR "training program*"))	551,352
5	1 and 2 and 3 and 4	1

Scopus

Search conducted: October 14, 2025

	Search	Results retrieved
1	TITLE-ABS-KEY (tuberculosis OR tb OR "mycobacterium tuberculosis" OR "pulmonary tb" OR ptb OR "latent tb" OR "mdr-tb" OR "xdr-tb")	469,725
2	TITLE-ABS-KEY ("health education" OR "health promotion" OR "patient education" OR "health literacy" OR "health communication" OR "behavior change communication" OR bcc OR "information education communication" OR iec OR "awareness campaign" OR "health advocacy" OR "health message" OR "community health education")	619,170
3	TITLE-ABS-KEY ("health personnel" OR "health worker" OR "community health worker" OR "frontline worker" OR "lay health worker" OR nurse OR "public health nurse" OR "tb educator" OR "tb coordinator" OR "outreach worker" OR counselor OR "health workforce" OR "healthcare provider")	838,220
4	TITLE-ABS-KEY ("professional competence" OR competence OR competency OR competencies OR skill OR "capacity building" OR "knowledge attitude practice" OR kap OR training OR "skill development" OR "performance improvement" OR proficiency OR qualification OR "core competency" OR empower OR ability)	6,140,464
5	1 and 2 and 3 and 4	290

Embase

Search conducted: October 13, 2025

	Search	Results retrieved
1	'tuberculosis'/exp OR tuberculosis OR tb OR 'mycobacterium tuberculosis' OR 'pulmonary tb' OR ptb OR 'latent tb' OR 'mdr-tb' OR 'xdr-tb'	487,339
2	'health education'/exp OR 'health promotion'/exp OR 'patient education'/exp OR 'health education' OR 'health promotion' OR 'patient education' OR 'health literacy' OR 'health communication' OR 'behavior change communication' OR bcc OR 'information education communication' OR iec OR 'awareness campaign' OR 'health advocacy' OR 'health message' OR 'community health education'	627,479
3	'health care personnel'/exp OR 'community health worker'/exp OR 'nurse'/exp OR 'allied health personnel'/exp OR 'health personnel' OR 'health worker' OR 'community health worker' OR 'frontline worker' OR 'lay health worker' OR nurse OR 'public health nurse' OR 'tb educator' OR 'tb coordinator' OR 'outreach worker' OR counselor OR 'health workforce' OR 'healthcare provider' OR 'health care provider' OR trainer OR facilitator	2,582,356
4	professional competence/exp OR 'clinical competence'/exp OR 'capacity building'/exp OR 'professional competence' OR competence OR competency OR competencies OR skill OR 'capacity building' OR 'knowledge attitude practice' OR kap OR training OR 'skill development' OR 'performance improvement' OR proficiency OR qualification OR 'core competency' OR empower OR ability OR 'competency framework' OR 'skill set' OR 'capacity strengthening'	3,069,896
5	1 and 2 and 3 and 4	424

CINAHL Complete (EBSCOhost)

Search conducted: October 13, 2025

	Search	Results retrieved
1	(MH "Tuberculosis+") OR (MH "Mycobacterium Tuberculosis") OR Tuberculosis OR TB OR "Mycobacterium tuberculosis" OR "pulmonary TB" OR PTB OR "Latent TB" OR "MDR-TB" OR "XDR-TB"	43,103

2	(MH "Health Education+") OR (MH "Health Promotion+") OR (MH "Patient Education+") OR "Health Education" OR "Health Promotion" OR "Patient Education" OR "health literacy" OR "Health Communication" OR "behavior change communication" OR BCC OR "Information Education Communication" OR IEC OR "awareness campaign" OR "health advocacy" OR "health message" OR "community health education"	281,067
3	(MH "Health Personnel+") OR (MH "Community Health Workers+") OR (MH "Nurses+") OR (MH "Allied Health Personnel+") OR (MH "Public Health Nurses") OR "health personnel" OR "health worker*" OR "community health worker*" OR "frontline worker*" OR "lay health worker*" OR nurse* OR "public health nurse" OR "tb educator*" OR "tb coordinator*" OR "outreach worker*" OR counselor* OR "health workforce" OR "healthcare provider*" OR "health care provider*" OR trainer* OR facilitator*	1,148,382
4	(MH "Professional Competence+") OR (MH "Clinical Competence+") OR (MH "Capacity Building") OR (MH "Staff Development") OR "professional competence" OR competenc* OR skill* OR "capacity building" OR "knowledge attitude practice" OR KAP OR training OR "skill development" OR "performance improvement" OR proficien* OR qualification* OR "core competenc*" OR empower* OR "ability" OR "competency framework" OR "skill set" OR "capacity strengthening"	739,043
5	1 and 2 and 3 and 4	95

Appendix II：Studies included in review

ID	Title	Authors	Year	Journal	Country
1	An approach to the diagnosis, treatment and referral of tuberculosis patients: The family practitioner's role	Ndjeka N.O. et al.	2008	S. Afr. Fam. Pract.	South Africa
2	An assessment of the HIV/TB knowledge and skills of home-based carers working in the North West province in South Africa	Engelbrecht J.G. et al.	2017	BMC Health Serv Res	South Africa
3	Facilitators and barriers to a hospital-based communication skills training programme: An interview study	Wolderslund M. et al.	2023	Int J Environ Res Public Health	Denmark
4	Health system challenges affecting HIV and tuberculosis integration at primary healthcare clinics in Durban, South Africa	Kalonji D., Mahomed O.H.	2019	Afr J Prim Health Care Fam Med	South Africa
5	Factors associated with health education delivery by rural doctors for tuberculosis patients in Shandong Province, China	Shi L. et al.	2010	Health Policy	China
6	A qualitative exploration of doctors and nurses experiences on the management of tuberculosis and HIV co-infection in a tuberculosis-HIV high burden community in northern KwaZulu-Natal, South Africa	Mabuza M.P., Shumba C.	2018	J Public Health Afr	South Africa
7	Before reaching the last mile' - Knowledge, attitude, practice and perceived barriers related to tuberculosis directly observed therapy among ASHA workers in Central India	Singh A.R. et al.	2017	J Epidemiol Glob Health	India
8	Community care worker perceptions of their roles in tuberculosis care and their information needs	Okeyo I., Dowse R.	2016	Health SA Gesondheid	South Africa
9	Community health workers' knowledge of Ubuntu informed care in tuberculosis, HIV, and AIDS in Gauteng province	Gundo R. et al.	2025	Curationis	South Africa
10	Distance learning course for healthcare professionals: Continuing education in tuberculosis	Cabral V.K. et al.	2017	Telemed J E Health	Brazil
11	Evaluating tuberculosis knowledge and awareness of effective control practices among health care workers in primary- and secondary-level medical institutions in Beijing, China	Zhang L. et al.	2024	BMC Infect Dis	China
12	Factors influencing health care workers' implementation of tuberculosis contact tracing in Kweneng, Botswana	Tlale L. et al.	2016	Pan Afr Med J	Botswana
13	Internal medicine residents' knowledge and practice of pulmonary tuberculosis diagnosis	Chida N. et al.	2018	Open Forum Infect Dis	United States
14	Bridging the gap: A strategic approach to upscale knowledge among diverse healthcare providers for effective tuberculosis management in Gujarat, India	Shah H. et al.	2024	Cureus	India
15	Managerial capacity of primary health care for tuberculosis control in different regions of Brazil	Villa T.C.S. et al.	2018	Texto Contexto Enferm	Brazil
16	Characteristics, knowledge, attitude, and practice of pharmacy personnel in supporting tuberculosis treatment: A multicenter cross-sectional study in a high-burden tuberculosis country	Pradipta I.S. et al.	2024	J Am Pharm Assoc	Indonesia
17	Community health workers in health systems strengthening: a qualitative evaluation from rural Haiti	Jerome G., Ivers L.C.	2010	AIDS	Haiti
18	Community perceptions of community health workers (CHWs) and their roles in management for HIV, tuberculosis and hypertension in Western	Rachlis B. et al.	2016	PLoS One	Kenya

	Kenya				
19	Enhancing the capacity of community health workers in prevention and control of epidemics and pandemics in Wakiso district, Uganda	Musoke D. et al.	2024	BMC Prim Care	Uganda
20	Entrustable professional activities (EPAs) for global health	Steeb D.R. et al.	2021	Acad Med	Multinational/Global
21	Competency building for lay health workers is an intangible force driving basic public health services in Southwest China	Liang S. et al.	2019	BMC Health Serv Res	China
22	Barriers and motivators affecting tuberculosis infection control practices of Russian health care workers	Woith W. et al.	2012	Int J Tuberc Lung Dis	Russia
23	Exploring strategies to improve the performance of community health volunteers for tuberculosis care and prevention: A qualitative study	Lukman M. et al.	2019	Int J Community Based Nurs Midwifery	Indonesia
24	Education on medically unexplained symptoms: a systematic review with a focus on cultural diversity and migrants	Mariman A. et al.	2023	Eur J Med Res	Multinational/Global
25	Changing professional practice in tuberculosis care: An educational intervention	Dick J. et al.	2004	J Adv Nurs	South Africa
26	Health education in rural communities with locally produced and locally relevant multimedia content	Molapo M., Marsden G.	2013	ACM International Conference Proceeding Series	South Africa
27	Effective communication approaches in tuberculosis control: Health workers' perceptions and experiences	Sriram S., Elangovan R.	2017	Indian J Tuberc	India
28	Knowledge and attitudes of tuberculosis management in San Juan de Lurigancho district of Lima, Peru	Kiefer E.M. et al.	2009	J Infect Dev Ctries	Peru
29	Knowledge and practice of health workers about control and prevention of multidrug-resistant tuberculosis in referral hospitals, Ethiopia	Alene K.A. et al.	2019	BMJ Open	Ethiopia
30	Knowledge, attitudes and practices of community health agents regarding pulmonary tuberculosis in a capital city in northeastern Brazil	Gaspar L. et al.	2019	Cien Saude Colet	Brazil
31	Piloting Siyakhana: A community health worker training to reduce substance use and depression stigma in South African HIV and TB care	Regenauer K.S. et al.	2024	PLOS Glob Public Health	South Africa
32	Prejudice and determinants regarding tuberculosis patients among medical students in Dalian, Northeast China	Yi Y. et al.	2024	Front Public Health	China
33	TB/HIV-related training, knowledge and attitudes of community health workers in the Free State province, South Africa	Heunis C. et al.	2013	Afr J AIDS Res	South Africa
34	Burden of 375 diseases and injuries, risk-attributable burden of 88 risk factors, and healthy life expectancy in 204 countries and territories, including 660 subnational locations, 1990-2023: a systematic analysis for the Global Burden of Disease Study 2023	GBD 2023 Risk Factor Collaborators	2025	Lancet	Global
35	Social multiplier effects: academics' and practitioners' perspective on the benefits of a tuberculosis operational research capacity-building program in Indonesia	Probandari A. et al.	2017	Glob Health Action	Indonesia
36	The use of a rating instrument to teach and assess communication skills of health-care workers in a clinic in the Western Cape	Steyn M. et al.	1999	Curationis	South Africa

Appendix III: Studies ineligible following full-text review

ID	Title	Authors
1	Do healthy doctors deliver better messages of health promotion to their patients?: Data from the SUN cohort study	Carlos S. et al.
2	Health and Nutrition Promotion Programs in Papua New Guinea: A Scoping Review	Chen J. et al.
3	Exploring the promise and reality of ward-based primary healthcare outreach teams conducting TB household contact tracing in three districts of South Africa	Chetty-Makkan C.M. et al.
4	Cost-effectiveness of educational outreach to primary care nurses to increase tuberculosis case detection and improve respiratory care: economic evaluation alongside a randomised trial	Fairall L. et al.
5	Perception Versus Reality: The Use of Teach Back by Medical Residents	Feinberg I. et al.
6	Exploring opportunities to strengthen rural tuberculosis health service delivery: a qualitative study with health workers in Tibet autonomous region, China	Haldane V. et al.
7	Geographical, social, and political contexts of tuberculosis control and intervention, as reported by mid-level health managers in Uganda	Johnson-Peretz J. et al.
8	Chinese health literacy scale for tuberculosis patients: a study on development and psychometric testing	Li Y. et al.
9	Community health workers and tuberculosis control: knowledge and perceptions	Maciel E.L. et al.
10	Our Healthier Nation: are general practitioners willing and able to deliver? A survey of attitudes to and involvement in health promotion and lifestyle counselling	McAvoy B.R. et al.
11	Eswatini Nursing Council Regulatory Reforms: Process towards Entry to Practice Examination	Msibi G. et al.
12	Community health care workers in pursuit of TB: Discourses and dilemmas	Onazi O. et al.
13	Midwives' perceptions and experiences of health promotion practice in Ghana	Owusu-Addo E.
14	Patients are not the same, so we cannot treat them the same - A qualitative content analysis of provider, patient and implementer perspectives on differentiated service delivery models for HIV treatment in South Africa	Pascoe S.J.S. et al.
15	Paediatricians' knowledge, attitudes and practice following provision of educational resources about prevention of prenatal alcohol exposure and Fetal Alcohol Spectrum Disorder	Payne J.M. et al.
16	Evaluation of lay health workers' needs to effectively support anti-tuberculosis treatment adherence in Malawi	Puchalski Ritchie L.M. et al.
17	Community health workers' knowledge on tuberculosis, control measures, and directly observed therapy	Rocha G.S. et al.
18	Patient perspectives on online health information and communication with doctors: a qualitative study of patients 50 years old and over	Silver M.P.
19	Health care providers' knowledge, attitude and perceived stigma regarding tuberculosis in a pastoralist community in Ethiopia: a cross-sectional study	Sima B.T. et al.
20	An integrated approach of community health worker support for HIV/AIDS and TB care in Angonia district, Mozambique	Simon S. et al.
21	MATRI-SUMAN' a capacity building and text messaging intervention to enhance maternal and child health service utilization among pregnant women from rural Nepal: study protocol for a cluster randomised controlled trial	Singh J.K. et al.

22	Nurse training to enhance adherence counselling for HIV-tuberculosis coinfection in South Africa: Integrative review	Ticha V. et al.
23	Assessment of doctors' knowledge regarding tuberculosis management in Lucknow, India: a public-private sector comparison	Vandan N. et al.
24	Counseling on lifestyle habits in the United States and Sweden: a report comparing primary care health professionals' perspectives on lifestyle counseling in terms of scope, importance and competence	Weinehall L. et al.

Appendix IV：Results after the scope review

Dimension	Category	Competency	Corresponding Reference ID
1 Professional Knowledge Base	1.1 Core TB knowledge	1.1.1 basic TB knowledge (etiology, transmission routes, affected organs)	[2], [31], [34]
		1.1.2 mastery of TB diagnostic criteria and procedures	[13]
		1.1.3 identification of TB risk factors	[13], [34]
		1.1.4 knowledge of TB drugs (types, mechanisms, side effects)	[2]
		1.1.5 TB treatment regimens (course, duration)	[2]
	1.2 Related domain knowledge	1.2.1 ability to integrate cross-disciplinary knowledge	[32]
		1.2.2 knowledge of disease associations (comorbidities, complications)	[31]
		1.2.3 understanding of program operations and health policies	[5], [34]
2 Communication and Health Education Skills	2.1 Core communication skills	2.1.1 general communication techniques	[1], [3], [21], [30]
		2.1.2 active listening	[1], [36]
		2.1.3 rapport building	[36]
		2.1.4 non-judgmental communication	[31], [32]
		2.1.5 empathy and emotional support	[23], [32]
		2.1.6 psychological support	[2], [36]
		2.1.7 patient validation	[36]
	2.2 Health education	2.2.1 patient education ability	[28], [29], [30]

	implementation skills		
		2.2.2 ability to develop health education plans	[2]
		2.2.3 ability to identify health education needs	[18], [26]
		2.2.4 information presentation and delivery	[8], [26]
		2.2.5 cognitive verification skills	[36]
	2.3 Special situation communication skills	2.3.1 ability to overcome barriers in discussing sensitive topics	[26]
		2.3.2 motivational interviewing skills	[31], [32]
		2.3.3 stigma response and anti-discrimination communication skills	[31], [32], [33]
	2.4 Educational methods/tools application	2.4.1 ability to use multimedia/digital tools	[14], [26], [27]
		2.4.2 ability to organize meetings and activities	[27]
		2.4.3 tool application and context adaptation ability	[36]
3 Clinical Practice and Problem-Solving Skills	3.1 Clinical practice skills	3.1.1 clinical decision-making	[13]
		3.1.2 general practical operation skills	[4], [13], [28], [29], [30]
		3.1.3 symptom recognition	[31]
		3.1.4 infection control implementation	[4]
	3.2 Problem-solving skills	3.2.1 general problem-solving	[31], [32], [36]
		3.2.2 practical problem analysis	[34]
		3.2.3 flexibility and adaptability	[28], [29]
		3.2.4 reflection and improvement skills	[33]
	3.3 Follow-up/case management skills	3.3.1 follow-up and tracking skills	[33]

		3.3.2 confidentiality management	[31]
		3.3.3 patient needs response ability	[36]
4 Professionalism and Personal Attributes	4.1 Professional values and motivation	4.1.1 sense of professional and ethical responsibility	[19], [22], [30], [33]
		4.1.2 sense of mission	[19], [22]
		4.1.3 motivation for self-improvement	[34], [35]
	4.2 Attitudes and affective traits	4.2.1 positive work attitude	[7], [16], [18], [24], [30]
		4.2.2 empathy and altruism	[8], [23]
		4.2.3 sense of achievement and pride	[8]
		4.2.4 work initiative	[8]
	4.3 Self-awareness and management	4.3.1 self-confidence	[19], [22]
		4.3.2 clarity of role perception	[17]
		4.3.3 self-emotion management	[31], [32]
		4.3.4 self-care	[31], [32]
		4.3.5 emotional stability	[32]
	4.4 Bias and stigma management	4.4.1 stigma awareness and anti-stigma literacy	[31], [32], [33], [35]
		4.4.2 ability to maintain objectivity	[32]
	4.5 Key personality traits	4.5.1 general personality traits (e.g., openness, conscientiousness)	[32], [35]
5 Training,Learning and Development competency	5.1 Training experiences and formats	5.1.1 formal training experience	[2], [5], [6], [7], [8], [14], [15], [16], [21]
		5.1.2 continuous training participation	[3], [8], [9], [10], [11], [12], [17], [18], [19], [20]

		5.1.3 standardized/specialized training	[4], [10], [23]
		5.1.4 distance education	[10]
		5.1.5 specific communication skills training	[19], [24]
	5.2 Learning and development ability	5.2.1 active learning ability	[6], [8]
		5.2.2 continuing education participation	[10], [14], [22]
		5.2.3 knowledge updating and continuous learning	[13], [28], [31], [33], [34]
		5.2.4 knowledge retention/updating	[8]
	5.3 Teaching and dissemination ability	5.3.1 teaching and training ability	[32], [35]
		5.3.2 knowledge transfer skills	[33]
		5.3.3 public education ability	[35]
6 Collaboration,Management and Resource Integration competency	6.1 Teamwork ability	6.1.1 communication and collaboration ability	[30], [35]
		6.1.2 professional consultation and collaboration	[13], [35]
		6.1.3 collaborative awareness	[33]
		6.1.4 networking ability	[34], [35]
	6.2 Resource integration and referral	6.2.1 resource linkage and referral ability	[31], [32], [34]
	6.3 Cross-sectoral coordination	6.3.1 cross-service communication and coordination ability	[4]
		6.3.2 task sharing and task shifting ability	[4]
	6.4 Community mobilization	6.4.1 community mobilization ability	[30]

		6.4.2 community trust-building ability	[26]
	6.5 Organizational/management ability	6.5.1 relationship management and communication environment creation	[18], [25]
		6.5.2 communication and persuasion skills	[26]
7 Research and Knowledge Translation Competence	7.1 Research ability	7.1.1 general knowledge and research skills	[35]
		7.1.2 operational research skills	[35]
	7.2 Knowledge translation ability	7.2.1 knowledge translation ability	[34], [35]
	7.3 Project innovation ability	7.3.1 project innovation ability	[34]
		7.3.2 targeted intervention design ability	[32]

Appendix V：Results after the first round of the Delphi method

Dimension	Category	Competency
1 Professional Knowledge Base	1.1 TB Medical Knowledge	1.1.1 Etiology and transmission mechanism of TB
		1.1.2 Clinical manifestations and classification of TB
		1.1.3 Diagnostic criteria and procedures for TB
		1.1.4 Knowledge of TB treatment regimens
		1.1.5 Knowledge of anti-TB drug side effects
	1.2 Epidemiological Knowledge	1.2.1 Identification of pulmonary TB risk factors
		1.2.2 Understanding of TB epidemic characteristics
	1.3 Policy and System Knowledge	1.3.1 National TB control policies
		1.3.2 Understanding of the health service system
2 Communication and Health Education Skills	2.1 Basic Communication Skills	2.1.1 Active listening ability
		2.1.2 Non-judgmental communication ability
		2.1.3 Empathy expression ability
		2.1.4 Clear expression ability
	2.2 Health Education Implementation Skills	2.2.1 Ability to assess health education needs
		2.2.2 Ability to develop health education plans
		2.2.3 Ability to develop health education materials
		2.2.4 Ability to evaluate educational outcomes
	2.3 Special Situation Communication Skills	2.3.1 Ability to communicate sensitive topics
		2.3.2 Motivational interviewing skills
		2.3.3 Psychological support and counseling ability
		2.3.4 Stigma response communication ability
	2.4 Educational Formats and Methods	2.4.1 Ability to use multimedia tools
		2.4.2 Ability to organize group activities

3 Clinical Practice and Problem-Solving Skills	3.1 Clinical Practice Skills	3.1.1 Symptom recognition and preliminary judgment ability
		3.1.2 Infection control implementation ability
		3.1.3 Follow-up management ability
	3.2 Problem-Solving Skills	3.2.1 Practical problem analysis ability
		3.2.2 Ability to handle emergency situations
		3.2.3 Reflection and improvement ability
	3.3 Case Management Skills	3.3.1 Confidentiality management ability
		3.3.2 Individualized intervention design ability
4 Professionalism and Self-Management	4.1 Professional Values	4.1.1 Sense of professional responsibility
		4.1.2 Sense of mission
		4.1.3 Altruistic spirit
	4.2 Work Attitude	4.2.1 Proactiveness
		4.2.2 Work enthusiasm and dedication
	4.3 Self-Awareness	4.3.1 Clarity of role perception
		4.3.2 Self-confidence
	4.4 Self-Management Skills	4.4.1 Emotional management ability
		4.4.2 Stress coping ability
		4.4.3 Self-care ability
5 Learning and Development competency	5.1 Learning Behaviors	5.1.1 Active learning behavior
		5.1.2 Continuous learning habit
	5.2 Training Participation	5.2.1 Formal training participation
		5.2.2 Continuing education participation
		5.2.3 Specialized skills training
	5.3 Knowledge Management Ability	5.3.1 Knowledge acquisition ability
		5.3.2 Knowledge updating ability
		5.3.3 Knowledge application ability

6 Collaboration and Resource Integration competency	6.1 Teamwork	6.1.1 Teamwork ability
		6.1.2 Interprofessional collaboration ability
	6.2 Resource Integration	6.2.1 Referral ability
		6.2.2 Resource linkage ability
	6.3 Cross-Sectoral Coordination	6.3.1 Inter-institutional communication ability
		6.3.2 Task coordination ability
	6.4 Community Work Ability	6.4.1 Community mobilization ability
		6.4.2 Community trust-building ability
7 Research and Innovation Literacy	7.1 Foundational Research Ability	7.1.1 Literature review ability
		7.1.2 Data collection ability
	7.2 Knowledge Translation Ability	7.2.1 Evidence-based practice ability
		7.2.2 Experience summarization ability
	7.3 Innovation and Improvement	7.3.1 Work improvement ability
		7.3.2 Intervention design ability

Appendix VI:PRISMA- ScR checklist

SECTION	ITEM	PRISMA-ScR CHECKLIST ITEM	REPORTED ON PAGE #
**TITLE**			
Title	1	Identify the report as a scoping review.	1
**ABSTRACT**			
Structured summary	2	Provide a structured summary that includes (as applicable): background, objectives, eligibility criteria, sources of evidence, charting methods, results, and conclusions that relate to the review questions and objectives.	1-2
**INTRODUCTION**			
Rationale	3	Describe the rationale for the review in the context of what is already known. Explain why the review questions/objectives lend themselves to a scoping review approach.	2
Objectives	4	Provide an explicit statement of the questions and objectives being addressed with reference to their key elements (e.g., population or participants, concepts, and context) or other relevant key elements used to conceptualize the review questions and/or objectives.	3
**METHODS**			
Protocol and registration	5	Indicate whether a review protocol exists; state if and where it can be accessed (e.g., a Web address); and if available, provide registration information, including the registration number.	3
Eligibility criteria	6	Specify characteristics of the sources of evidence used as eligibility criteria (e.g., years considered, language, and publication status), and provide a rationale.	3
Information sources*	7	Describe all information sources in the search (e.g., databases with dates of coverage and contact with authors to identify additional sources), as well as the date the most recent search was executed.	4
Search	8	Present the full electronic search strategy for at least 1 database, including any limits used, such that it could be repeated.	4,Appendix I: Search strategy

SECTION	ITEM	PRISMA-ScR CHECKLIST ITEM	REPORTED ON PAGE #
Selection of sources of evidence†	9	State the process for selecting sources of evidence (i.e., screening and eligibility) included in the scoping review.	5
Data charting process‡	10	Describe the methods of charting data from the included sources of evidence (e.g., calibrated forms or forms that have been tested by the team before their use, and whether data charting was done independently or in duplicate) and any processes for obtaining and confirming data from investigators.	5
Data items	11	List and define all variables for which data were sought and any assumptions and simplifications made.	5
Critical appraisal of individual sources of evidence§	12	If done, provide a rationale for conducting a critical appraisal of included sources of evidence; describe the methods used and how this information was used in any data synthesis (if appropriate).	Not applicable
Synthesis of results	13	Describe the methods of handling and summarizing the data that were charted.	5
**RESULTS**			
Selection of sources of evidence	14	Give numbers of sources of evidence screened, assessed for eligibility, and included in the review, with reasons for exclusions at each stage, ideally using a flow diagram.	7-9
Characteristics of sources of evidence	15	For each source of evidence, present characteristics for which data were charted and provide the citations.	7-8
Critical appraisal within sources of evidence	16	If done, present data on critical appraisal of included sources of evidence (see item 12).	Not applicable
Results of individual sources of evidence	17	For each included source of evidence, present the relevant data that were charted that relate to the review questions and objectives.	Appendix II：Studies included in review
Synthesis of results	18	Summarize and/or present the charting results as they relate to the review questions and objectives.	22-24
**DISCUSSION**			
Summary of evidence	19	Summarize the main results (including an overview of concepts, themes, and types of evidence available), link to the review questions and objectives, and consider the relevance to key groups.	11-13

SECTION	ITEM	PRISMA-ScR CHECKLIST ITEM	REPORTED ON PAGE #
Limitations	20	Discuss the limitations of the scoping review process.	14
Conclusions	21	Provide a general interpretation of the results with respect to the review questions and objectives, as well as potential implications and/or next steps.	15
**FUNDING**			
Funding	22	Describe sources of funding for the included sources of evidence, as well as sources of funding for the scoping review. Describe the role of the funders of the scoping review.	19
